# Guidelines for reporting on animal fecal transplantation (GRAFT) studies: recommendations from a systematic review of murine transplantation protocols

**DOI:** 10.1080/19490976.2021.1979878

**Published:** 2021-09-29

**Authors:** Kate R. Secombe, Ghanyah H. Al-Qadami, Courtney B. Subramaniam, Joanne M. Bowen, Jacqui Scott, Ysabella Z.A. Van Sebille, Matthew Snelson, Caitlin Cowan, Gerard Clarke, Cassandra E. Gheorghe, John F. Cryan, Hannah R. Wardill

**Affiliations:** a School of Biomedicine, Adelaide Medical School, University of Adelaide, Adelaide, SA, Australia; b Precision Medicine Theme (Cancer), South Australian Health and Medical Research Institute, Adelaide, SA, Australia; c UniSA Online, University of South Australia, Adelaide, SA, Australia; d Department of Diabetes, Central Clinical School, Monash University, Melbourne, VIC, Australia; e School of Psychology and Brain and Mind Centre, University of Sydney, Sydney, NSW, Australia; f Department of Psychiatry and Neurobehavioural Science, Department of Anatomy and Neuroscience, and APC Microbiome Ireland, University College Cork, Cork, Ireland; g Department of Psychiatry and Neurobehavioural Science and APC Microbiome Ireland, University College Cork, Cork, Ireland; h Department of Anatomy and Neuroscience and APC Microbiome Ireland, University College Cork, Cork, Ireland

**Keywords:** Fecal microbiome transplantation, FMT, reporting guidelines, replication, reproducibility, methodology, guidelines

## Abstract

Fecal microbiota transplant (FMT) is a powerful tool used to connect changes in gut microbial composition with a variety of disease states and pathologies. While FMT enables potential causal relationships to be identified, the experimental details reported in preclinical FMT protocols are highly inconsistent and/or incomplete. This limitation reflects a current lack of authoritative guidance on reporting standards that would facilitate replication efforts and ultimately reproducible science. We therefore systematically reviewed all FMT protocols used in mouse models with the goal of formulating recommendations on the reporting of preclinical FMT protocols. Search strategies were applied across three databases (PubMed, EMBASE, and Ovid Medline) until June 30, 2020. Data related to donor attributes, stool collection, processing/storage, recipient preparation, administration, and quality control were extracted. A total of 1753 papers were identified, with 241 identified for data extraction and analysis. Of the papers included, 92.5% reported a positive outcome with FMT intervention. However, the vast majority of studies failed to address core methodological aspects including the use of anaerobic conditions (91.7% of papers lacked information), storage (49.4%), homogenization (33.6%), concentration (31.5%), volume (19.9%) and administration route (5.3%). To address these reporting limitations, we developed the*Guidelines for Reporting Animal Fecal Transplant (GRAFT)* that guide reporting standards for preclinical FMT. The GRAFT recommendations will enable robust reporting of preclinical FMT design, and facilitate high-quality peer review, improving the rigor and translation of knowledge gained through preclinical FMT studies.

## Introduction

The collection of microorganisms in the gastrointestinal tract, termed the gut microbiota, is growing in appreciation for its dynamic regulation of host function and disease. While large-scale sequencing studies have provided unprecedented insight into the range of conditions associated with the microbiome, they are unable to provide conclusive evidence for how the microbiota causally contributes to disease and how it can be exploited to modify disease risk or progression.^1^

Fecal microbiota transplantation (FMT) is a powerful technique in which the microbial community is transferred from a donor to a recipient host, transferring a unique microbial enterotype to prevent, treat or (preclinically) induce disease, or modulate host physiology. Clinically, FMT is now second-line therapy for antibiotic-resistant *Clostridioides difficile* (*C. difficile*) and its scope is expanding.^2^ Indeed, there is a growing list of emerging indications under investigation in a variety of preclinical models and pilot cohorts.^3,4^ In addition to its therapeutic application, preclinical FMT is increasingly used to dissect causal microbiota-dependent mechanisms and understand how unique microbial profiles dictate disease risk.

Although a powerful technique, the regulatory landscape for clinical use of FMT is challenging, largely due to the ambiguities regarding its classification (i.e. biological product equivalent to blood or organ versus drug).^5^ Despite this, there are clear recommendations and guidelines for FMT preparation, administration, and quality control when used in human recipients.^6^ In contrast, preclinical FMT protocols vary enormously, as recently highlighted,^7^ with little to no recommendations on best practice and reporting standards. This profoundly hinders the ability to interpret and replicate preclinical FMT studies and the inconsistent application of experimental approaches compromises clinical translation.

The need for better guidance of preclinical FMT protocols is underscored by the additional layers of complexity that are introduced in a preclinical setting. For example, experimental design, preparation and administration are complicated by the coprophagic nature of rodents. While some studies have exploited this behavior (co-housing to induce microbial transfer),^8^ there is significant variability in how this technique is applied and the omission of key methodological detail hinders experimental replication, thus undermining subsequent translation.^9^ Similarly, while bowel preparation is recommended for colonoscopically administered FMT in humans, the necessity for an appropriate equivalent in recipient rodents remains unclear.

Germ-free (GF, i.e. those without any resident microorganisms) mice have often been used as recipients in FMT models, as their lack of existing gut microbiota represents a highly effective ‘take-up’ of the donor FMT. However, as previously highlighted,^10^ barriers related to cost and logistics have prevented widespread use of this model, and concerns regarding how closely they mimic normal immune development have plagued interpretation of results generated.^11^ Therefore, antibiotic-induced depletion of the microbiota has become common practice to ablate the microbial community of the gut. However, there are vast differences in the antibiotic treatment specifications used in animal models, including FMT. These include type, dose and duration, which can introduce significant variability in ablative capacity, with persisting pathogens confounding results (for a comprehensive review of this topic, please see Gheorghe et al.^12^).

While antibiotic treatment represents a particularly common area of variability, in reality, each step of preclinical FMT protocols can introduce bias. This was recently highlighted by Walter et al. (2020) who identified that 95% of preclinical FMT studies reported successful transfer of the clinical phenotype to the recipient rodent – a figure deemed implausible by the authors.^13^ While these findings also reflect publication biases, they underscore the need to advocate for standardization of approaches for preclinical FMT when inferring causality to prevent unrealistic expectations that may undermine the credibility of microbiome science and delay its translation.

A key element of this enhanced rigor must be clarity in the methodological standards and reporting to improve consistency and transparency within the field, both of which will strengthen the reproducibility of findings. As such, we systematically reviewed published literature on preclinical FMT use in mice to provide a snapshot of current reporting patterns and, in collaboration with key microbiome research sites and networks, developed a set of minimum reporting guidelines for future preclinical FMT studies.

## Results and discussion

Study Selection

**Figure 1.**
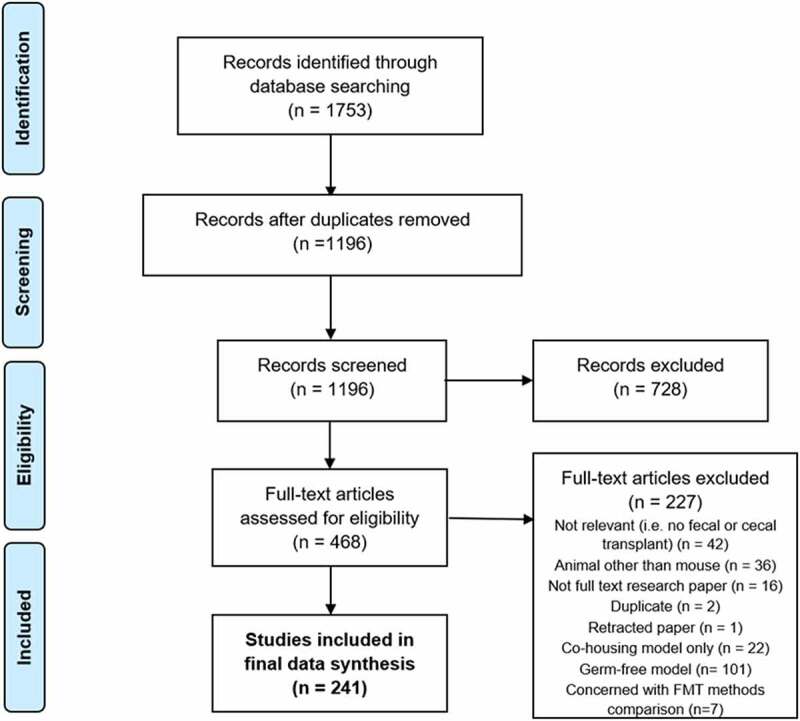
PRISMA flow chart for identification and selection of eligible studies

Of 1753 screened studies, a total of 241 were included. One thousand one hundred and ninety-six were screened via title and abstract, with 728 excluded as not relevant. Four hundred and sixty-eight full-text articles were assessed. Two hundred and twenty-seven were excluded at the full-text stage (Figure 1).

Study Characteristics

**Figure 2.**
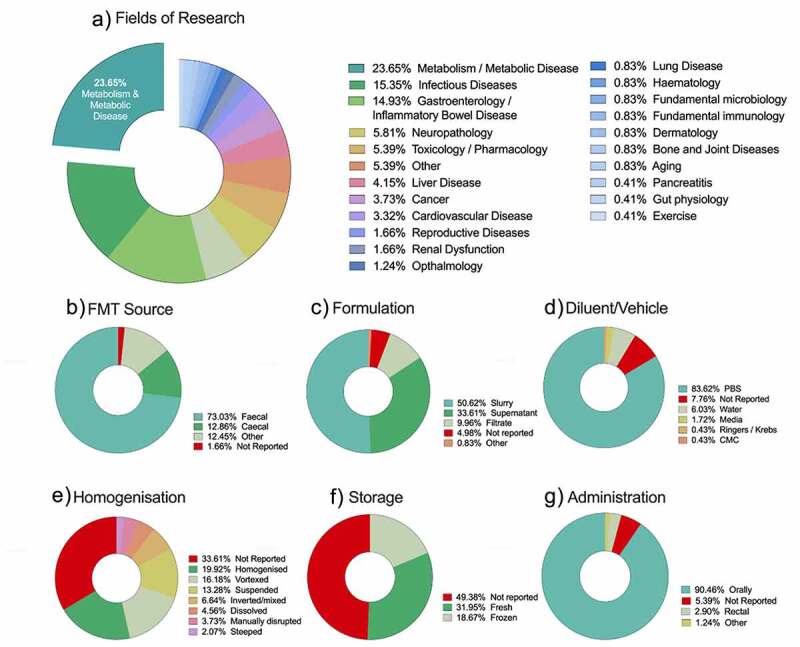
Key data extracted from N = 241 studies included for analysis

We studied papers evaluating FMT across a range of indications (Figure 2a). The most common area of investigation was metabolism/metabolic disease, accounting for 23.7% of all papers reviewed. Other areas of investigation included infectious diseases (15.4%), gastroenterology/inflammatory bowel disease (14.9%) and cognition/behavior/affect disorders (6.6%).

Studies ranged in the sample size used per experimental group (median [range]:8 [2–70]), reflecting varying power requirements for specific models. Disappointingly, 21.4% of the eligible studies did not clearly state the sample size of the recipient group. While the sample size for the donor FMT group was not extracted in our analysis due to low levels of reporting, it is also important to acknowledge that this should also be clearly reported alongside information on whether FMT contents are pooled across multiple donor samples. Donor sample size is particularly pertinent in the use of human donors, as recently outlined by Gheorghe et al. with the use of a single donor considered N = 1^12^.

## Data extracted

### Collection, processing and storage

There are several aspects of FMT preparation that must be acknowledged and highly protocolized for rigorous results: collection of donor stool, processing and storage. The vast majority of papers used fecal pellets to prepare the FMT product (73.0%; Figure 2b), with the remaining using cecal (12.9%) contents or other gastrointestinal products (e.g. duodenal aspirates and feces, mucosal scrapings, small and large intestinal contents collected from a culled mouse). A range of preparation techniques were used to produce the FMT, including filtrates, supernatants, and slurries. In the papers reviewed, a fecal slurry was most commonly used (50.6%; Figure 2c), with supernatants and filtered products used in 33.6% and 9.9% of papers, respectively.

In any FMT preparation, the vehicle/diluent must be carefully considered. In the studies included for analysis, phosphate buffered saline (PBS) was the most common solution (80.5%; Figure 2d), with a small number of studies including additives to the PBS (glycerol, cysteine hydrochloride) to improve microbial viability. The concentration of cysteine-hydrochloride was consistent across all studies (0.05%), whilst the concentration of glycerol ranged from 5% to 50%. While only 7.5% of studies failed to report the vehicle solution used for FMT preparation, 91.7% of studies failed to report whether this solution was reduced (i.e. de-oxygenated) or if the FMT was prepared under anaerobic conditions.

The high number of studies that failed to explicitly state whether FMT was prepared under anaerobic conditions is concerning as it has been reported that FMT prepared under aerobic conditions profoundly decreases microbial viability, altering microbial metabolite synthesis and abundance of many anaerobic commensals.^14–16^ Similarly, the way in which the fecal/cecal contents were processed was poorly reported, with 33.6% of studies failing to report on homogenization. This methodological step was generally reported with limited detail, using broad terminology such as “dissolved”, “mixed” or “suspended” (Figure 2e), with only 12% of studies providing sufficient detail for replication of the homogenization step. A similar observation was made for filtration methods used when preparing supernatants or bacterial preparations, with 31.5% of studies failing to report on any filtration or “clean up” steps. For clarity and replication, manual filtration should be defined by the size of the strainer used and centrifugation defined using standard metrics (*x g*, min, ^o^C).

Once processed, the final FMT product can and should be quantified in terms of its concentration. Strikingly, 31.5% of studies failed to report a concentration, with the remaining studies using a wide range of units, including mg/ml (63.0%), pellets/ml (13.9%) and CFU/ml (9.6%). While we do not intend on recommending a specific unit to define concentration, it is critical that the final FMT product is defined in a standard unit of measurement that can be replicated by others. Studies reporting pellets (but no volume) or milliliters (but no weight) were deemed irreproducible.

The final FMT product can then be used immediately (fresh) or stored and used at a later date. As such, clarity on this methodological detail must be clearly provided particularly in light of the evidence that shows storage conditions impact microbial preservation and viability.^17,18^ Of the papers included in our analysis, 31.9% administered freshly prepared (i.e. not stored) FMT. A number of papers (18.7%) noted that the FMT product was frozen (−20°C and −80°C) prior to administration; however, close to half of the papers (49.4%) did not report any methodological detail on storage conditions (figure 2f).

### Recipient preparation and FMT administration

Once the FMT has been prepared, there are many considerations in its administration related to both the product itself and the recipient, including typical reporting standards related to husbandry. Of the studies included, 30.2% failed to provide any detail on animal housing conditions (i.e. single vs co-housed). Given the coprophagic nature of rodents, it is critical that this be clearly reported in all studies in which FMT is used to acknowledge/exclude potential confounding impacts.

**Figure 3.**
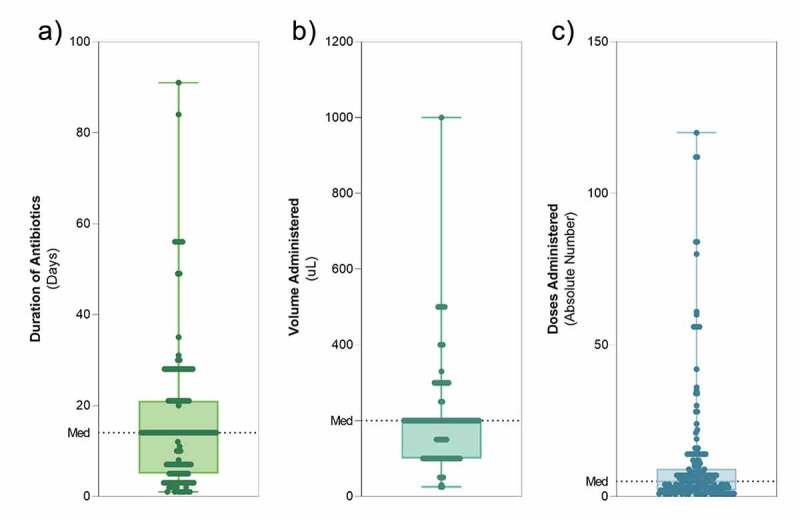
Distribution of objective FMT-related variables across N = 241 studies included. A) days of antibiotic exposure, B) volume of FMT administered, C) number of FMT doses administered. All data are shown as individual studies with median/range. Median: A = 14, B = 200, C = 5

We also investigated how, if at all, recipient mice were prepared for FMT. As suggested previously,^19^ there is some evidence that bowel lavage or cleansing could improve FMT efficacy; however, these remain speculative and not widely recommended. Accordingly, very few studies (N = 3) included in our analysis reported bowel preparation procedures in recipient mice. One study fasted mice the night prior to FMT administration and two studies provided PEG3350 as a laxative beforehand. Antibiotic-induced depletion was used in 60.5% of studies, most commonly administered in drinking water (61.4%) for a median of 14 days [1–91 range] (Figure 3a). The most common combination was a cocktail of ampicillin, neomycin, vancomycin, and metronidazole (ANVM; Table S1).

While antibiotic-induced depletion has, to date, been an area of critical methodological consideration in optimal FMT administration, it remains an area of contentious debate. In fact, increasing evidence suggests that antibiotic depletion may not be necessary for FMT uptake; albeit the evidence is conflicting. While Ji et al. (2017) reported great FMT durability with antibiotic depletion compared to either a MoviPrep bowel cleanse or no pretreatment,^20^ others have shown no difference. For example, Freitag et al. (2019) showed that pre-treatment with antibiotics did not improve the overall engraftment of the donor microbiome, and only improved the engraftment of a small number of taxa.^19^ While Ji et al. (2017, *not included in this review*) utilized a human donor microbiome, Freitag et al. (2019) used a mouse donor microbiome, suggesting that antibiotic administration may be useful in improving FMT engraftment across the species barrier. While we do not intend on recommending the use of antibiotics in FMT studies, these findings highlight the need to clearly describe all pre-treatments used to prepare the recipient for FMT.

FMT administration can be achieved via oral or rectal routes. It has been previously speculated that as oral gavage inoculum needs to pass through the acidic stomach environment, rectal administration may be more efficient.^21^ However, a previous study of FMT in mice showed that specific pathogen-free mice treated with antibiotics and then orally or rectally inoculated with donor mice gut microbiota had no differences in microbial community after inoculation.^22^ As such, while rectal administration is preferable in efficacy and safety outcomes for clinical FMT use, oral gavage is often selected in mouse studies, presumably due to convenience.^23^ In line with these findings, the overwhelming majority of studies included in our analysis opted for oral administration (90.4%; Figure 2g) and only a small number used rectal administration (2.9%). Three studies reported alternative methods of administration, including directly pipetting into the oropharynx,^24^ which can be used when oral gavage is not permitted (note that other methods including co-housing and vertical transfer (generational transfer between mothers and pups) were excluded). The route of administration was the most consistently reported aspect of FMT methodology; only 5.3% of papers either did not report or did not clearly state how their FMT product was administered.

Administration volume is also critical to FMT replication, with our analyses identifying a large range of volumes administered to recipient mice (median[range]: 200 µl [25–1000]; Figure 3b). Volume was not reported in 19.9% of studies included in our analysis. Similarly, the frequency of FMT (or absolute number of treatments) was not reported in 13.2% of studies. Of the studies that did report this metric, there was again a significant range (1–120 treatments) with a median of 5 FMT treatments (Figure 3c).

In administering the FMT, adequate control procedures must be implemented to account for the impact of the procedure. This can be achieved by administering an autologous FMT or one prepared using sham/control animals. Alternatively, the vehicle solution can be administered. Forty percent of papers included in our analysis failed to use a control arm or report on what their control animals received. Of the studies that did report this detail, 56% used FMT prepared from sham/control animals and 34% used the vehicle solution.

### Quality control and uptake confirmation/durability

The success of FMT relies on a number of complex and interacting factors, but central to its general efficacy is its viability (after collection and processing) and uptake (“durability”).

We defined quality control (QC) as analysis of the FMT product before administration to the recipient, i.e. to identify the presence of potential pathogens and confirm viability of the product. No information regarding quality control was reported in 88.4% of studies. Of the few studies that did include QC, 16S rRNA sequencing was the most commonly used technique (53.6%) followed by standard culture (32.1%). Given the inability of 16S rRNA sequencing to determine the viability of the microbial community, these findings underscore the need to implement standardized preclinical FMT guidelines to ensure appropriate QC is incorporated at project inception.

Confirming uptake of the FMT is also critical to its efficacy. Le Roy et al. (2018) defined the durability of the FMT procedure as: 1. Establishment of high levels of bacterial taxa from the inoculum in recipients, 2. Relative abundance of bacterial taxa as similar as possible in the inoculum and recipients, and 3. The removal of a high amount of endogenous bacterial taxa in non-GF recipients.^7^ This can be determined by microbial analysis of the FMT inoculum, and gut microbial contents of the recipient both before and after FMT occurs.

Overall, explicit reference to durability assessment was lacking with microbial analyses often reported in the study but rarely compared between the FMT donor, product, and recipient. In fact, 22.1% of papers did not report or did not confirm uptake of the FMT in any way. Of the papers that did report, 16S rRNA sequencing was the primary method (86.9%) with other studies reporting culture- (6.0%) or PCR-based approaches (4.3%).

### Reproducibility and rigor

A recent systematic review searched scientific literature for studies suggesting a causal relationship between an altered human microbiome and disease or physiological condition.^13^ Of the papers meeting the inclusion criteria, all but two (95%), suggested that fecal transfer from diseased donors resulted in a disease phenotype. Due to the wide range and complexity of pathologies studied in these papers, the authors suggested that the causal claims seem unlikely across this wide range. Similarly, in our study, we found that 92.5% of papers showed that FMT had an effect. This may reflect publication bias – a tendency to favor positive findings for publication, or it could also suggest that the introduction of any new complex combination of microbes via FMT may cause a protective immune response in the gut, that manifests as a change in symptoms or disease severity. Thus demonstrating the wide variety of potential uses for FMT. Regardless, as suggested by Walter et al. (2020), microbiome science would benefit from increased rigor and critique.^13^ A key part of this scientific rigor is transparent and reproducible methodology.^13,25^

**Figure 4.**
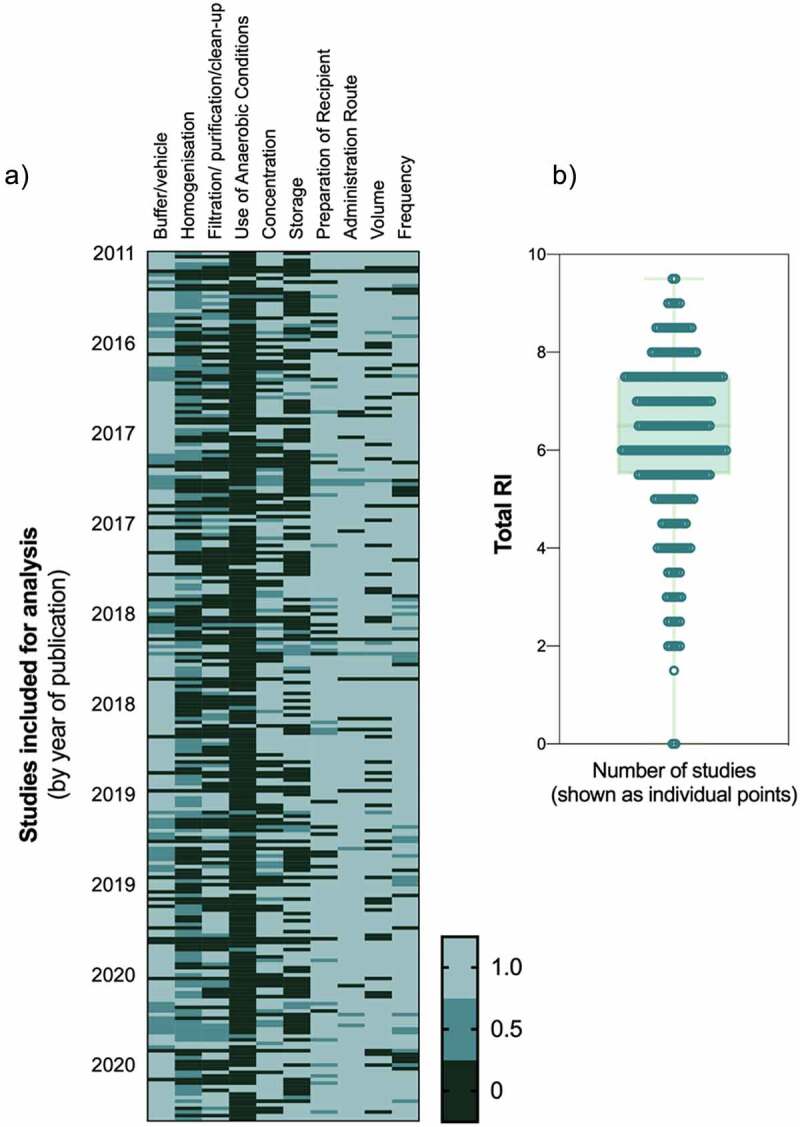
Reproducibility index assessment. Each study was assessed against 10 criteria where 0 = not reported, 0.5 = reported with insufficient detail or 1 = reported with sufficient detail for replication. A) heatmap of individual scores for all N = 241 studies, B) total reproducibility index (RI) for all studies with median ± IQR. The median score was 6.5

Throughout our analysis, we found that many methods described in published manuscripts did not have sufficient detail to be completely replicated. Therefore, we developed a reproducibility index containing 10 key aspects of FMT methodology and assigned a score from 0 to 1 for each variable, where 0 = not reported, 0.5 = reported with insufficient detail and 1 = reported with sufficient detail for replication (Figure 4). The median total value of this index was 6.5, with 23.6% of papers gaining a total value of 5 or more. Two papers had a reproducibility index of 9.5/10 in our review, the highest observed. The first paper (Krisko et al., 2020) paid particular detail to the preparation of donor mice, and the use of anaerobic conditions in FMT preparation.^26^ The second paper (Zhou et al., 2019) provided good detail about the amount, and concentration of the administered FMT product.^27^ In addition, Foligne et al. clearly outlined group numbers of both donors and recipient mice.^28^ While this measure provides an objective assessment of the level of detail in reporting, it is important to recognize that this should be interpreted with caution as the index is not validated. Thorough review of the literature yielded no appropriate method for assessing methodological reporting in this way, and as such, the reproducibility index was developed specifically for this study.

## The GRAFT recommendations and future steps

Our systematic review revealed an overall lack of clarity in the reporting of FMT methods. In almost all variables we investigated, there was not only a lack of consistency in FMT protocols, but also a lack of clarity and detail in methodological reporting. For example, for FMT concentration, as well as the actual concentration ranging widely, 7 different units were used to report this key step in FMT preparation. These findings point to a lack of authoritative guidance on preclinical FMT studies for both authors and reviewers.

**Figure 5.**
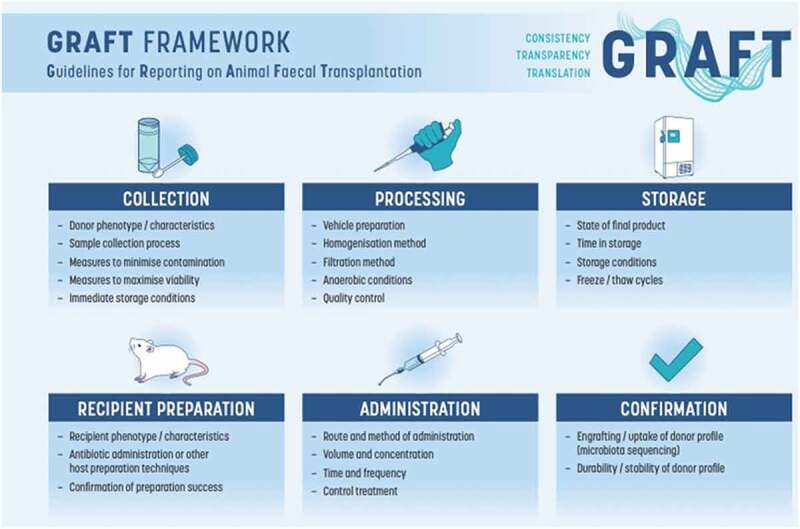
GRAFT framework for reproducible and transparent reporting

Due to the low level of detail found in many papers and the low mean score from our reproducibility index scoring, we suggest that a minimum set of reporting standards for preclinical FMT studies would be useful. As such, we present here the GRAFT (Guidelines for Reporting on Animal Fecal Transplantation) recommendations (Table 1, Figure 5) along with a simple checklist (File S1) that can be used at project inception/design, manuscript preparation, and review. By providing these recommendations, we hope to increase the transparency and reproducibility of preclinical FMT procedures, thus elevating their translational strength. While our systematic review intentionally restricted our search to studies with mice, we argue that, given the similarity in FMT procedures across species, the GRAFT recommendations can also be used to guide FMT use in other species, and may offer guidance in human-animal transplantation if followed in combination with the recommendations presented by Walter et al. (2020).^13^

**Table 1.** GRAFT guidelines for reporting animal fecal transplant studies

Collection	
Donor phenotype/characteristics	a. Number of individual donors (per group) b. Detailed description of donor characteristics (see also ARRIVE Guidelines for animal donors), including but not limited to: - Species/strain of donors - Sex/gender of donors - Age and developmental stage of donors c. Details of control and experimental phenotypes (e.g. healthy vs disease phenotype) - Inclusion and exclusion criteria, with particular attention to factors relevant to the microbiome (e.g. diet, exercise) d. Details on housing and husbandry - Facility specifications (i.e. SPF/GF; If GF, include specifications of animal unit/isolator) - Co-housing vs single-housing - Arrangement of cages across racks (particularly with regards to separation of donor groups and separation from FMT recipient animals) - Bedding and chow
Sample collection process	a. Type of sample (i.e. fecal pellet, intestinal/cecal content, mucosal scraping) b. Time of day of collection and details on minimization of circadian rhythm effects c. Animal handling during collection d. Details on sample collection methods (e.g. placing animal into clean cage until defecation or direct postmortem collection from cecum or intestines) - HUMAN DONORS: collection methods (e.g. normal defecation or directly from specific region of intestines during colonoscopy, medically indicated or otherwise)
Measures to minimize contamination	a. Aseptic procedures and protocols adopted during and after collection
Immediate storage conditions	a. Methods to minimize oxidative stress (i.e. use of transport medium) b. Immediate storage conditions (e.g. stored in reduced medium, snap frozen in liquid nitrogen, kept on ice or at ambient temperature etc.) c. Details on pooling of samples (if relevant) - Method of pooling (e.g. equal weight of initial sample from each donor prior to processing or equal volume of processed liquid) - Number of individual donors within each pool
Processing	
Vehicle preparation	a. Details of solution, including formulation, concentration, pH, temperature, volume b. Additives used to support microbial viability c. If de-oxygenated solution is used, specify method of de-oxygenation
Concentration	a. Report using standardized units (mg/ml) - Avoid inaccurate units (e.g. pellets/ml)
Homogenization method	a. Equipment used (e.g. vortex, Stomacher, autoclaved spatula) b. Intensity (using standardized units where possible) c. Time and temperature
Filtration method	a. Method of filtration (e.g. gravity, centrifuge, strainer, stomacher bag) - Centrifuge: specify time, *x g* and temperature - Gravity: specify time and conditions (i.e. ambient, anaerobic, temperature) - Physical strainer/membrane: specify pore size or equivalent detail and filtration method
Anaerobic conditions	a. Clearly state if/when anaerobic conditions were used b. Details of anaerobic conditions (i.e. chamber type, gas mix, temperature etc.)
Quality control	a. Method used to assess FMT quality and composition prior to administration (e.g. plating, genomic sequencing) b. Outcome of quality assessment (e.g. CFU/ml, diversity index)
Storage	
State of final product	a. Define administered product as: - Fecal slurry (i.e. fecal contents with minimal filtration) – or - - Fecal supernatant/filtrate (i.e. microbial free) – or - - Microbial preparation (i.e. lyophilized or other)
Time in storage	a. Time between preparation of final product and administration
Storage conditions	a. Details of storage conditions between preparation and administration, including: - Volume per aliquot - Storage temperature - Duration of storage b. If fecal product is used fresh, this must be clearly stated with details including: - Short term storage conditions (i.e. on ice, fridge, room temperature, anaerobic chamber) - Time between preparation and administration
Freeze/thaw cycles	a. Method of thawing fecal product prior to administration - Include number of freeze-thaw cycles
Recipient preparation	
Recipient phenotype/characteristics	a. Number of recipient animals (per group) - If multiple animals receive FMT from the same donor (or pooled sample), this number should be reported for each donor, separately to the total b. Detailed description of recipient characteristics (see also ARRIVE Guidelines), including but not limited to: - Species/strain of recipients - Sex of recipients - Age and developmental stage of recipients c. Details on housing and husbandry - Facility specifications (i.e. SPF/GF; If GF, specifications of animal unit/isolator) - Co-housing vs single-housing - Arrangement of cages across racks (particularly with regards to separation of experimental groups and separation from FMT donor animals) - Bedding and chow
Host preparation techniques	a. Methods of host preparation used prior to transplantation (e.g. antibiotic depletion, bowel cleansing, fasting) with relevant detail, including but not limited to: - Duration - Frequency (e.g. of changing antibiotic solution) - Specific treatment used (e.g. antibiotic names and concentrations) b. Preparation methods used in control group(s), with details as above c. Adverse events in response to preparation treatment (e.g. weight loss with antibiotics)
Confirmation of preparation success	a. Ideally, successful depletion of recipient microbiota should be confirmed through fecal analysis prior to FMT
Administration	
Route and method of administration	a. Oral or rectal administration (or both) b. Method of administration (e.g. oral gavage, lavage, enema, coprophagia) c. Details on use of anesthesia or fasting prior to administration (particularly rectal) and coprophagic approaches (i.e. was additional FMT smeared on coat to improve uptake)
Volume and concentration	a. Define in standard units for each individual FMT - Specify if absolute unit or relative to body weight of recipient
Time and frequency	a. Time of day of administration b. Frequency of FMT, including total number and daily frequency (i.e. a total of 3 FMT by oral gavage at a frequency of 1 per day, number of days between doses) c. Time between FMT administration and assessment of outcomes (i.e. disease status, behavioral change, microbiota composition etc.)
Control treatment	a. Define treatment received by control animals (e.g. vehicle solution, autologous transplant, heat-killed FMT, FMT from control donor group) - Include control formulation, concentration, volume, time, and frequency as above
Confirmation	
Engrafting/uptake of donor profile	a. Define how engraftment/uptake of the FMT procedure was confirmed (e.g. 16S rRNA/shotgun sequencing, fecal culture) - It is recommended that the same analysis be applied to the final FMT product administered to compare composition of donor and recipient b. Timing of sample collection for engraftment assessment relative to FMT administration and outcome assessments c. Details on sample collection methods, as for donor: - Time of day of collection - Handling during collection - Method: Placing animal into clean cage until defecation or direct postmortem collection from colon, cecum or other site
Durability/stability of donor profile	a. Particularly for lengthy experimental designs, it may be informative to analyze the recipient microbiota at multiple time-points after FMT administration to determine the long-term stability of the donor profile within the recipient

While these guidelines provide the much-needed structure for preclinical FMT protocols, it is critical to emphasize that we do not aim to recommend what methods should be used, as different experimental endpoints and research questions will clearly need a different methodological design (as previously discussed by Gheorghe et al.^12^). However, by consistently reporting the following set of guidelines, future studies will be more reproducible and thus be more likely to generate clinically relevant outcomes. Similarly, these guidelines will facilitate and structure the peer review process for preclinical FMT studies, which based on our analyses is poorly guided. We envisage that the GRAFT reporting recommendations will facilitate interpretation and experimental replication in future preclinical FMT studies, improving reproducibility, allowing better systematic review and meta-analysis, and future guidance on the most optimal methods to answer specific scientific questions.

## Conclusions

This systematic review aimed to determine the most common protocols for FMT experiments in mice. Our key overarching finding was that many of the details required to reproduce these protocols were missing from the majority of papers, leading to the development of our minimum set of reporting guidelines. In the future, we urge researchers to clearly outline their protocols in order to provide transparency, increase reproducibility, and ultimately enhance the chances of producing clinically relevant and translatable knowledge.

## Key findings:

- 92.5% of studies included reported a positive outcome of FMT intervention
- Fecal slurries, containing both the microbiota and their metabolome, were the most common form of FMT product
- 91.7% of studies did not report on the use of anaerobic conditions during FMT product preparation
- Method of homogenization was not referred to in 33.6% of studies
- 49.4% did not report storage conditions for FMT product
- 21.4% of studies did not report on the sample size of the recipient group
- Antibiotic-induced depletion was the most common form of recipient preparation
- 5.3% of studies did not describe how the FMT was administered
- 19.9% did not report the volume of FMT administered
- FMT durability/uptake was not confirmed in 22.1% of studies
- 88.4% of studies did not perform any quality control
- 40% of studies did not report on control FMT conditions
- The vast majority of studies were deemed irreproducible

## Methods

### Focus question

This systematic review aimed to answer the question: “what FMT protocols are being used in preclinical mouse models of health and disease?” FMT protocols were then used to define core aspects of preclinical FMT methodology and develop a set of minimum reporting standards.

### Study design

The protocol for this systematic review was conducted in accordance with the Preferred Reporting Items for Systematic Reviews and Meta-Analysis (PRISMA) guidelines.^29^

### Search strategy

We completed a comprehensive search using the electronic databases PubMed, Ovid Medline and Embase on June 30, 2020 (no date restrictions). The search parameters were tailored to each database, and the full search string for each database can be found in the Supplementary Information. We searched for papers including fecal or cecal material transplant. In total, 1753 papers were identified from our database search.

### Selection criteria

Two reviewers (KRS and HRW) conducted the initial literature search and removed duplicate articles. Following this, entries from prior to 2010 were removed to ensure only modern FMT protocols were included. Initially, abstracts and titles were screened using Covidence systematic review software web program to assess eligibility (Veritas Health Innovation, Melbourne, Australia). Available at www.covidence.org). After this abstract screen, full-text articles were again assessed by the same reviewers. Two hundred and forty-one articles were selected for data extraction.

We aimed to retrieve only full-text, peer-reviewed, original experimental studies performed in mice. Studies must have been published in English. To be included in the review, studies must have completed a fecal or cecal microbiota transplant where mice were both the donor and the recipient.

Studies were excluded if they: used human or other non-mice microbial material for transplant, utilized GF mice as recipients or utilized a co-housing only approach to FMT. Secondary studies such as review papers, methodological protocols and conference abstracts were also excluded.

### Data extraction and analysis

Seven reviewers (KRS, HRW, GHA, CBS, JS, MS, CC) independently extracted relevant information from the selected papers using standard data collection templates. We extracted all available methodological data on FMT from the main paper or Supplementary Information. Key information included: donor and recipient characteristics (age, strain, antibiotic use), FMT preparation and storage methods, FMT administration (dosage, number of treatments, administration route) and use of quality control methods. To quantify the reproducibility of preclinical FMT protocols included in our analysis, we developed a reproducibility index based on 10 variables of preclinical FMT, irrespective of model or study goals. The criteria were as follows: buffer/vehicle, method of homogenization, filtration steps, storage (if applicable), concentration of final FMT product, pre-conditioning of the recipient, route of administration, volume administered, frequency of administration and the inclusion of anaerobic conditions. Reviewers marked each criterion as 0 = not reported, 0.5 = mentioned, 1 = mentioned with appropriate detail (to be able to effectively replicate the study). Importantly, studies were assessed based on whether these parameters were *reported*, not for *how* they were performed. This index was not developed to provide a statistically comprehensive measure of reproducibility, and as such there was not necessarily a linear relationship between the score and overall reproducibility of the study.

## Supplementary Material

Supplemental Material

## Data Availability

The data that support the findings of this study are openly available in Figshare at https://doi.org/10.25909/14978817.v2.

## Declarations

Ethics approval and consent to participate: Not applicable.

Consent for publication: Not applicable.

## References

[1] Kho ZY, Lal SK. The human gut microbiome - a potential controller of wellness and disease. Front Microbiol. 2018;9:1835. doi:10.3389/fmicb.2018.01835. 30154767 PMC6102370

[2] Green JE, Davis JA, Berk M, Hair C, Loughman A, Castle D, Athan E, Nierenberg AA, Cryan JF, Jacka F, Marx W. Efficacy and safety of fecal microbiota transplantation for the treatment of diseases other than Clostridium difficile infection: a systematic review and meta-analysis. Gut Microbes. 2020 Nov 9;12(1):1–14. doi:10.1080/19490976.2020.1854640. PMC7757860 33345703

[3] Allegretti JR, Mullish BH, Kelly C, Fischer M. The evolution of the use of faecal microbiota transplantation and emerging therapeutic indications. Lancet (London, England). 2019 Aug 3;394(10196):420–431. doi:10.1016/s0140-6736(19)31266-8. 31379333

[4] Wortelboer K, Nieuwdorp M, Herrema H. Fecal microbiota transplantation beyond Clostridioides difficile infections. EBioMedicine. 2019 Jun;44:716–729. doi:10.1016/j.ebiom.2019.05.066. 31201141 PMC6606746

[5] Hoffmann DE, Palumbo FB, Ravel J, Rowthorn V, von Rosenvinge E. A proposed definition of microbiota transplantation for regulatory purposes. Gut Microbes. 2017 May 4;8(3):208–213. doi:10.1080/19490976.2017.1293223. 28318396 PMC5479380

[6] Burz SD, Abraham AL, Fonseca F, David O, Chapron A, Béguet-Crespel F, Cénard S, Le Roux K, Patrascu O, Levenez F, et al. A guide for ex vivo handling and storage of stool samples intended for fecal microbiota transplantation. Sci Rep. 2019 Jun 20;9(1):8897. doi:10.1038/s41598-019-45173-4. 31222022 PMC6586871

[7] Le Roy T, Debédat J, Marquet F, Da-Cunha C, Ichou F, Guerre-Millo M, Kapel N, Aron-Wisnewsky J, Clément K. Comparative evaluation of microbiota engraftment following fecal microbiota transfer in mice models: age, kinetic and microbial status matter. Front Microbiol. 2018;9:3289. doi:10.3389/fmicb.2018.03289. 30692975 PMC6339881

[8] Ridaura VK, Faith JJ, Rey FE, Cheng J, Duncan AE, Kau AL, Griffin NW, Lombard V, Henrissat B, Bain JR, Muehlbauer MJ, et al. Gut microbiota from twins discordant for obesity modulate metabolism in mice. Science (New York, NY). 2013;341(6150):1241214. doi:10.1126/science.1241214. PMC3829625 24009397

[9] Morais LH, Golubeva AV, Moloney GM, et al. Enduring behavioral effects induced by birth by caesarean section in the mouse. Curr Biol. 2020 Oct 5;30(19):3761–3774 e6. doi:10.1016/j.cub.2020.07.044. 32822606

[10] Lundberg R, Toft MF, August B, Hansen AK, Hansen CHF. Antibiotic-treated versus germ-free rodents for microbiota transplantation studies. Gut Microbes. 2016;7(1):68–74. doi:10.1080/19490976.2015.1127463. 26744774 PMC4856451

[11] Spichak S, Guzzetta KE, O’Leary OF, Clarke G, Dinan TG, Cryan JF. Without a bug’s life: germ-free rodents to interrogate microbiota-gut-neuroimmune interactions. Drug Discov Today. 2018;28:14.

[12] Gheorghe CE, Ritz NL, Martin JA, Wardill HR, Cryan JF, Clarke G. Investigating causality with fecal microbiota transplantation in rodents: applications, recommendations and pitfalls. Gut Microbes. 2021 01/01 2021;13(1):1941711. doi:10.1080/19490976.2021.1941711. 34328058 PMC8331043

[13] Walter J, Armet AM, Finlay BB, Shanahan F. Establishing or exaggerating causality for the gut microbiome: lessons from human microbiota-associated rodents. Cell. 2020 Jan 23;180(2):221–232. doi:10.1016/j.cell.2019.12.025. 31978342

[14] Costello SP, Hughes PA, Waters O, et al. Effect of fecal microbiota transplantation on 8-week remission in patients with ulcerative colitis: a randomized clinical trial. JAMA. 2019;321(2):156–164. doi:10.1001/jama.2018.20046. 30644982 PMC6439766

[15] Ng SC, Kamm MA, Yeoh YK, et al. Scientific frontiers in faecal microbiota transplantation: joint document of Asia-Pacific Association of Gastroenterology (APAGE) and asia-pacific society for digestive endoscopy (APSDE). Gut. 2020;69(1):83–91. doi:10.1136/gutjnl-2019-319407. 31611298 PMC6943253

[16] Papanicolas LE, Choo JM, Wang Y, et al. Bacterial viability in faecal transplants: which bacteria survive? EBioMedicine. 2019 03/01/ 2019;41:509–516. doi:10.1016/j.ebiom.2019.02.023. 30796005 PMC6444077

[17] Takahashi M, Ishikawa D, Sasaki T, et al. Faecal freezing preservation period influences colonization ability for faecal microbiota transplantation. J Appl Microbiol. 2019;126(3):973–984. doi:10.1111/jam.14167. 30489686

[18] Dorsaz S, Charretier Y, Girard M, et al. Changes in microbiota profiles after prolonged frozen storage of stool suspensions. Front Cell Infect Microbiol. 2020;10:77. doi:10.3389/fcimb.2020.00077. 32185143 PMC7058979

[19] Freitag TL, Hartikainen A, Jouhten H, et al. Minor effect of antibiotic pre-treatment on the engraftment of donor microbiota in fecal transplantation in mice. Front Microbiol. 2019;10:2685. doi:10.3389/fmicb.2019.02685. 31824463 PMC6881239

[20] Ji SK, Yan H, Jiang T, et al. Preparing the gut with antibiotics enhances gut microbiota reprogramming efficiency by promoting xenomicrobiota colonization. Front Microbiol. 2017;8:1208. doi:10.3389/fmicb.2017.01208. 28702022 PMC5487471

[21] Hu J, Chen L, Tang Y, et al. Standardized preparation for fecal microbiota transplantation in pigs. Front Microbiol. 2018;9:1328. doi:10.3389/fmicb.2018.01328. 29971061 PMC6018536

[22] Lützhøft DO, Sánchez-Alcoholado L, Tougaard P, et al. Short communication: gut microbial colonization of the mouse colon using faecal transfer was equally effective when comparing rectal inoculation and oral inoculation based on 16S rRNA sequencing. Res Vet Sci. 2019;126:227–232. doi:10.1016/j.rvsc.2019.09.009. 31627163

[23] Park JC, Im S-H. Of men in mice: the development and application of a humanized gnotobiotic mouse model for microbiome therapeutics. Exp Mol Med. 2020 09/01 2020;52(9):1383–1396. doi:10.1038/s12276-020-0473-2. 32908211 PMC8080820

[24] KS J, MT B, CS K, KY K. Antibiotics impair murine hematopoiesis by depleting the intestinal microbiota. Blood. 2017;129(6):729–739. doi:10.1182/blood-2016-03-708594. 27879260 PMC5301822

[25] Brüssow H. Problems with the concept of gut microbiota dysbiosis. Microb Biotechnol. 2020 Mar;13(2):423–434. doi:10.1111/1751-7915.13479. 31448542 PMC7017827

[26] Krisko TI, Nicholls HT, Bare CJ, et al. Dissociation of adaptive thermogenesis from glucose homeostasis in microbiome-deficient mice. Cell Metab. 2020;31(3):592–604.e9. doi:10.1016/j.cmet.2020.01.012. 32084379 PMC7888548

[27] Zhou ZL, Jia XB, Sun MF, et al. Neuroprotection of fasting mimicking diet on mptp-induced parkinson’s disease mice via gut microbiota and metabolites. Neurotherapeutics. 2019;16(3):741–760. doi:10.1007/s13311-019-00719-2. 30815845 PMC6694382

[28] Foligné B, Plé C, Titécat M, et al. Contribution of the gut microbiota in P28GST-mediated anti-inflammatory effects: experimental and clinical insights. Cells. 2019;8(6):577. doi:10.3390/cells8060577. PMC6627314 31212833

[29] Moher D, Liberati A, Tetzlaff J, Altman DG, Group P. Preferred reporting items for systematic reviews and meta-analyses: the PRISMA statement. BMJ. 2009 Jul 21;339(jul21 1):b2535. doi:10.1136/bmj.b2535. 19622551 PMC2714657

