# Supplementary material for: Guidelines for reporting on animal fecal transplantation (GRAFT) studies: recommendations from a systematic review of murine transplantation protocols
Source: Gut Microbes. 2021 Sep 29;13(1):1979878. doi: 10.1080/19490976.2021.1979878 (PMC8489962; doi:10.1080/19490976.2021.1979878)
Supplement: Supplemental Material [file KGMI_A_1979878_SM0222.docx]

**Supplementary material**

Supplementary methods

*Search Strategy*

Rat studies were initially included in the search strategy and were removed at a later date due to a refinement of the original research topic.

Ovid Medline:

(Fecal OR faecal OR cecal OR caecal OR microbi*).ti,ab adj5 (Transplant* OR transfer* OR engraftment OR innocul*).ti,ab AND (Mice OR mouse OR rat OR rats OR murine).ti,ab

PubMed:

"Fecal Microbiota Transplantation"[mh] OR Fecal microbiota transplant[tiab] OR Fecal microbiota transfer[tiab] OR Fecal microbiota engraftment[tiab] OR Fecal microbiota transplant[tiab] OR Fecal microbiota transfer[tiab] OR Fecal microbiota engraftment[tiab] OR fecal microbiota inoculum[tiab]  OR faecal microbiota inoculum[tiab] OR microbiome transplant[tiab] OR microbiome transfer[tiab] OR microbiome engraftment[tiab] OR microbiome transplant[tiab] OR microbiome transfer[tiab] OR microbiome engraftment[tiab] OR microbiome inoculum[tiab]  OR microbiome inoculum[tiab] OR cecal microbiota transplant[tiab] OR cecal microbiota transfer[tiab] OR cecal microbiota engraftment[tiab] OR caecal microbiota transplant[tiab] OR caecal microbiota transfer[tiab] OR caecal microbiota engraftment[tiab] OR cecal microbiota inoculum[tiab] OR caecal microbiota inoculum[tiab] AND Mice[mh] OR mice[tiab] OR mouse[tiab] OR murine[tiab] OR rats[mh] OR rat[tiab]

EMBASE:

“Fecal Microbiota Transplantation”/exp OR ‘Fecal microbiota transplant’:ti,ab OR ‘Fecal microbiota transfer’:ti,ab OR ‘Fecal microbiota engraftment’:ti,ab OR ‘Fecal microbiota transplant’:ti,ab OR ‘Fecal microbiota transfer’:ti,ab OR ‘Fecal microbiota engraftment’:ti,ab OR ‘fecal microbiota inoculum’:ti,ab  OR ‘faecal microbiota inoculum’:ti,ab OR ‘microbiome transplant’:ti,ab OR ‘microbiome transfer’:ti,ab OR ‘microbiome engraftment’:ti,ab OR ‘microbiome transplant’:ti,ab OR ‘microbiome transfer’:ti,ab OR ‘microbiome engraftment’:ti,ab OR ‘microbiome inoculum’:ti,ab  OR ‘microbiome inoculum’:ti,ab OR ‘cecal microbiota transplant’:ti,ab OR ‘cecal microbiota transfer’:ti,ab OR ‘cecal microbiota engraftment’:ti,ab OR ‘caecal microbiota transplant’:ti,ab OR ‘caecal microbiota transfer’:ti,ab OR ‘caecal microbiota engraftment’:ti,ab OR ‘cecal microbiota inoculum’:ti,ab OR ‘caecal microbiota inoculum’:ti,ab AND “Mouse”/exp OR mice:ti,ab OR mouse:ti,ab OR murine:ti,ab OR “rat”/exp OR rat:ti,ab

Supplementary table

| **Table S1: Details of antibiotic-induced microbial depletion** | | |
| --- | --- | --- |
| Route of administration | Gavage | 34 |
|  | Drinking water | 91 |
|  | Both | 8 |
|  | Not reported | 11 |
|  | Unclear | 3 |
|  |  |  |
| Cocktail vs single | Cocktail | 128 |
|  | Single | 17 |
|  | Not reported | 2 |
|  |  |  |
| Most common single agent | Streptomycin | 8 |
| Most common combination | ANVM cocktail | 74 |
|  |  |  |
| Duration (days) | Unclear/ not reported | 6 |
|  | Min | 1 |
|  | 25 percentile | 5 |
|  | Median | 14 |
|  | 75 percentile | 21 |
|  | Max | 91 |
|  | Range | 90 |
|  | Mean | 15.69 |
|  |  |  |
| Antibiotics used | Total | 22 |
|  | Average number used per study | 3.69 |
|  | Vancomycin | 113 |
|  | Ampicillin | 109 |
|  | Metronidazole | 108 |
|  | Neomycin | 95 |
|  | Streptomycin | 24 |
|  | Gentamicin | 21 |
|  | Ciprofloxacin | 19 |
|  | Imipenem | 9 |
|  | Amphotericin | 8 |
|  | Penicillin | 8 |
|  | Colistin | 7 |
|  | Kanamycin | 4 |
|  | Amoxicillin | 3 |
|  | Ceftazidime | 3 |
|  | Primaxin | 2 |
|  | Bacitracin | 2 |
|  | Ceftriaxone | 1 |
|  | Polymycin/Polymyxin | 1 |
|  | Carbenicillin | 1 |
|  | Trimethoprim | 1 |
|  | Clindamycin | 1 |
|  | Nystatin | 1 |
